# Melatonin alleviates sepsis-induced acute lung injury by inhibiting necroptosis via reducing circulating mtDNA release

**DOI:** 10.1186/s10020-025-01228-z

**Published:** 2025-05-07

**Authors:** Yuce Peng, Jia Xu, Lingyu Wei, Minghao Luo, Shenglong Chen, Xuebiao Wei, Suxin Luo, Zedazhong Su, Zhonghua Wang

**Affiliations:** 1 Department of Geriatrics, Guangdong Provincial Geriatrics Institute, Guangdong Provincial People’s Hospital, Guangdong Academy of Medical Sciences, Southern Medical University, Guangzhou, China; 2 Department of Geriatric Cardiology, The First Affiliated Hospital of Kunming Medical University, Kunming, China; 3 Department of Cardiology, the First Affiliated Hospital of Chongqing Medical University, Chongqing, China; 4 Department of emergency, The first affiliated hospital of Sun Yat-sen University, Guangzhou, China; 5 Department of Cardiology, Guangdong Provincial People’s Hospital, Guangdong Cardiovascular Institute, Guangdong Academy of Medical Sciences, Southern Medical University, Guangzhou, China; 6 Department of Critical Care Medicine, Guangdong Provincial People’s Hospital (Guangdong Academy of Medical Sciences), Southern Medical University, Guangdong, China; 7 Department of Geriatric Intensive Medicine, Guangdong Provincial Geriatrics Institute, Guangdong Provincial People’s Hospital, Guangdong Academy of Medical Sciences, Southern Medical University, Guangdong, China; 8 Guangdong Provincial Geriatrics Institute, Guangdong Provincial People’s Hospital, Guangdong Academy of Medical Sciences, Southern Medical University, Guangzhou, China

**Keywords:** Melatonin, Sepsis, ALI, Necroptosis, mtDNA-STING

## Abstract

**Background:**

Sepsis is a life-threatening condition that often leads to severe complications, including acute lung injury (ALI), which carries high morbidity and mortality in critically ill patients. Melatonin (Mel) has shown significant protective effects against sepsis-induced ALI, but its precise mechanism remains unclear.

**Methods:**

A cecal ligation and puncture (CLP) model was used to induce sepsis in male C57BL/6 mice, which were divided into four groups: Control, Sham, CLP, and CLP + Mel. ALI severity was evaluated via hematoxylin and eosin (H&E) staining, lung wet/dry ratio, and serum biomarkers (SP-D, sRAGE). Inflammatory cytokines (IL-1β, IL-6, TNF-α) were measured in serum and bronchoalveolar lavage fluid using ELISA. Circulating mitochondrial DNA (mtDNA) subtypes (D-loop, mt-CO1, mMito) were quantified by real-time PCR. TUNEL staining was performed to assess lung cell apoptosis. Necroptosis and STING pathway activation were analyzed via Western blot and immunofluorescence.

**Results:**

Sepsis led to increased circulating mtDNA levels and activation of necroptosis signaling pathways. Melatonin treatment alleviated sepsis-induced ALI, improving survival, reducing inflammatory cytokines and mtDNA release, and suppressing necroptosis. Intraperitoneal injection of mtDNA in mice activated necroptosis, while RIP1 inhibitor Nec-1 counteracted mtDNA-induced lung damage and necroptosis in sepsis-induced ALI. Additionally, melatonin significantly inhibited STING pathway activation. Further experiments revealed that STING modulation influenced necroptosis protein expression and mediated melatonin’s protective effects in sepsis-induced ALI.

**Conclusion:**

Melatonin mitigates sepsis-induced ALI by suppressing necroptosis through inhibition of STING activation and reduction of mtDNA release. These findings suggest melatonin as a potential therapeutic strategy for sepsis-induced ALI.

## Introduction

Sepsis is a life-threatening condition characterized by organ dysfunction due to an uncontrolled immune response to infection (Fleischmann et al. 2016; Singer et al. 2016), and is one of the major causes of death in developed countries (Shankar-Hari et al. 2016). According to data released by the Global Burden of Disease Study, there were an estimated 48.9 million cases and 11 million deaths worldwide in 2017 (Rudd et al. 2020). A systematic review of 166,479 patients noted that the septic shock-associated crude mortality was 46.5% (95% CI, 42.7-50.3%) (Shankar-Hari et al. 2016). Among the affected organs, the respiratory system is particularly vulnerable, because of inflammation, microthrombosis and innate immune activation caused by sepsis, and approximately 50% of patients with severe sepsis will develop acute lung injury (ALI) (Marcos-Ramiro et al. 2014; Lane et al. 1988; Sevransky et al. 2004), which may deteriorate into acute respiratory distress syndrome (ARDS) and respiratory failure (Bowdish et al. 2023). Previous studies have demonstrated that pro-inflammatory cytokines and oxidative stress contribute to alveolar-capillary barrier disruption, exacerbating ALI progression (Ning et al. 2022; Alsabani et al. 2022; Hou et al. 2021). However, the precise molecular mechanisms underlying sepsis-induced ALI remain poorly understood, necessitating further research to develop effective therapeutic strategies.

Necroptosis, a regulated form of necrotic cell death, differs from apoptosis and is characterized by the release of pro-inflammatory mediators, dysregulated DNA hydrolysis, and cytoplasmic content leakage (Gao et al. 2022; Pasparakis and Vandenabeele 2015; Tong et al. 2022). Receptor-interacting serine/threonine protein kinase-3 (RIPK3), a key mediator of necroptosis, is activated through interactions with receptor-interacting serine/threonine protein kinase-1 (RIPK1), and mixed lineage kinase domain-like protein (MLKL), leading to necrotic cell death (Linkermann et al. 2012; Galluzzi et al. 2017). Recent studies suggest that necroptosis plays a critical role in ALI, as secondary necroptosis contributes to damage-associated molecular patterns (DAMPs), membrane rupture, and excessive lung inflammation, ultimately promoting cell death, tissue injury, and organ failure (Sauler et al. 2019; Hao et al. 2023). Inhibition of necroptosis has been shown to alleviate lipopolysaccharide (LPS)-induced lung injury, indicating that targeting necroptosis could be a promising strategy for treating sepsis-induced ALI (Shashaty et al. 2019).

Mitochondria play a vital role in metabolism, energy production, and immune responses(West et al. 2015). During necroptosis, mitochondrial dysfunction exacerbates tissue damage, with mitochondrial DNA (mtDNA) released into the extracellular environment, triggering inflammatory signaling pathways (Maeda and Fadeel 2014). The cytosolic DNA sensor cyclic GMP-AMP synthase (cGAS) detects exogenous or endogenous DNA fragments and activates the stimulator of interferon genes (STING) pathway (Wang et al. 2020a; Gao et al. 2013). STING, an innate immune adaptor protein, promotes the activation of interferon regulatory factor 3 (IRF3) and nuclear factor-κB (NF-κB), leading to increased expression of pro-inflammatory cytokines (Barber 2015). Studies have shown that aberrant STING activation is closely linked to ALI pathogenesis (Hu et al. 2019; Zhang et al. 2023). However, the specific relationship between sepsis-induced ALI, mtDNA release, and STING activation remains unclear.

Melatonin (N-acetyl-5-methoxytryptamine) is a hormone synthesized by the pineal gland that regulates circadian rhythms (Cipolla-Neto et al. 2014; Yuge et al. 2020). Beyond its role in sleep regulation, melatonin has demonstrated protective effects in various systems, including the respiratory, cardiovascular, tumor immune, and central nervous systems (Reiter et al. 2016). Melatonin exerts antioxidant effects by scavenging free radicals and enhancing endogenous antioxidant enzyme activity. It also plays a role in maintaining mitochondrial dynamics by regulating mitochondrial fusion and fission balance (Yao et al. 2023). Pi et al. reported that melatonin protects mitochondria through a SIRT3-dependent mechanism, reducing inflammation-induced damage in sepsis by enhancing superoxide dismutase 2 (SOD2) activity (Pi et al. 2015). Additionally, Zhou et al. demonstrated that melatonin alleviates myocardial ischemia-reperfusion injury by inhibiting necroptosis (Zhou et al. 2018), while Sun et al. found that melatonin-mitochondrial combination therapy significantly reduced ALI severity in rats (Sun et al. 2015). However, the precise mechanisms through which melatonin mitigates ALI remain unclear. In this study, we aimed to investigate the role of necroptosis in sepsis-induced lung injury and evaluate the protective effects of melatonin. Specifically, we explored whether melatonin attenuates necroptosis by modulating mtDNA release and inhibiting STING activation, providing insights into its potential therapeutic application in sepsis-induced ALI.

## Methods

### Animals and ethics

Healthy male C57BL/6 mice (8 weeks old, 20–22 g) were purchased from the Chongqing Medical University Animal Center. The mice were housed under a 12-hour light/dark cycle with ad libitum access to food and water in a specific pathogen-free (SPF) barrier system. A total of 183 mice were used in this study. All experimental procedures followed the National Institutes of Health (NIH) guidelines and were approved by the Animal Ethics Committee of Guangdong Provincial People’s Hospital.

### Cecal ligation and puncture (CLP) model

A sepsis-induced ALI model was established using the cecal ligation and puncture (CLP) method as previously described. Briefly, mice were anesthetized with 2% isoflurane, and a midabdominal incision was made to expose the cecum. The distal ileocecal junction was ligated with a 3 − 0 suture and punctured with a 20-gauge needle to ensure smooth stool extrusion. The cecum was then repositioned into the abdominal cavity, and the muscle and skin layers were sutured separately. Postoperatively, mice received a subcutaneous injection of 1 mL of 37℃ saline and were kept at 37℃ until fully recovered. The mice were then returned to their cages and closely monitored.

### Experimental groups and treatments

For survival analysis, mouse mortality was recorded every 6 h, and animals in critical condition were euthanized. To assess sepsis progression, six mice were randomly sacrificed every 6 h post-CLP for blood, bronchoalveolar lavage fluid (BALF), and lung tissue collection. For treatment evaluation, mice were randomly assigned to 12 experimental groups. Following CLP, the experimental groups received intraperitoneal injections of either melatonin (30 mg/kg), DMXAA (10 mg/kg), H151 (10 mg/kg), mtDNA (5 mg/kg), or Nec-1 (1 mg/kg). The control group received an equivalent volume of vehicle consisting of 10% DMSO and 90% 20% SBE-β-CD in saline. Mice were euthanized 24 h post-CLP, and blood, BALF, and lung tissue were collected for analysis. A full list of antibodies and chemical reagents is provided in Table 1.

**Table 1:** Antibody and chemical reagents

Category	Company	Catalog No
**Antibody**		
RIP1	Affinity Biosciences	AF7877
p-RIP1(Ser166)	Affinity Biosciences	AF2398
RIP3	Affinity Biosciences	DF10141
p-RIP3(Ser232)	Affinity Biosciences	AF7443
MLKL	Proteintech	66,675
p-MLKL(Ser358)	Affinity Biosciences	AF7420
STING	Proteintech	19,851
TBK1	Proteintech	67,211
p-TBK1(Ser172)	Proteintech	82,383
P65	Cell Signal Technology	8242 S
p-P65(Ser536)	Cell Signal Technology	3033 S
IRF3	Proteintech	11,312
p-IRF3(Ser396)	Affinity Biosciences	AF2436
β-Actin	Proteintech	66,009
**Chemical reagents**		
Mouse IL-1β ELISA KIT	Solarbio	SEKM-0002
Mouse IL-6 ELISA KIT	Solarbio	SEKM-0007
Mouse TNF-α ELISA KIT	Solarbio	SEKM-0034
Mouse Surfactant protein D ELISA KIT	Solarbio	SEKM-0196
Mouse RAGE ELISA KIT	Solarbio	SEKM-0128
Mouse HMGB1 ELISA KIT	Solarbio	SEKM-0145
Mouse MIP-2 ELISA KIT	Solarbio	SEKM-0114
H151	MedChemExpress	HY-112,693
DMXAA	MedChemExpress	HY-10,964
Nec-1	MedChemExpress	HY-15,760

### Bronchoalveolar lavage fluid (BALF) collection

Mice were euthanized via carbon dioxide asphyxiation. The trachea was exposed, and a sterile cannula was inserted. BALF was collected by flushing the airway three times with 1 mL of PBS. Total protein concentration in BALF was measured using a bicinchoninic acid (BCA) protein assay kit (Beyotime).

### Lung wet/dry ratio

Lung edema was assessed by measuring the lung wet/dry weight ratio as previously described (Matute-Bello et al. 2011). The lungs were excised immediately after euthanasia and weighed (wet weight). The tissue was then dried at 60℃, and the dry weight was recorded once it stabilized. The wet/dry ratio was calculated as wet weight/dry weight.

### Histological analysis

The left lung was excised, fixed in 4% paraformaldehyde, and dehydrated using graded ethanol (70–100%). Tissues were embedded in paraffin, sectioned at 5 μm thickness, and stained with hematoxylin and eosin (H&E) for histopathological evaluation. Lung injury was scored based on a standardized scale (Table 2)(Kang et al. 2022).

**Table 2:** Lung injury score indicators

Parameter	Score per field		
	0	1	2
A. Neutrophils in the alveolar space	None	1–5	> 5
B. Neutrophils in the interstitial space	None	1–5	> 5
C. Hyaline membranes	None	1	> 1
D. Proteinaceous Debris filling the airspaces	None	1	> 1
E. Alveolar septal thickening	< 2×	2×-4×	> 4×

Score=[(20 × A) + (14 × B) + (7 × C) + (7 × D) + (2 × E)] / (number of fields ×100)

### ELISA for inflammatory markers

Blood samples were collected from the posterior orbital venous plexus, centrifuged at 3000 rpm for 10 min, and serum was extracted. Levels of IL-1β, IL-6, TNF-α, MIP-2, and HMGB1 in serum and BALF were measured using ELISA kits according to the manufacturer’s instructions.

### MtDNA isolation and quantification

Circulating mtDNA was extracted from serum using the QIAmp DNA Mini Kit (QIAGEN, #51304) per the manufacturer’s instructions. DNA concentration and purity were assessed using a NanoDrop 2000 (Thermo Fisher Scientific, Waltham, MA, USA). Quantification of mtDNA levels was performed via real-time polymerase chain reaction (qPCR) using primer sequences listed in Table 3. 18 S ribosomal RNA was amplified as an internal reference gene. Primer sequences were listed in Supplementary Data2. The 2^−ΔΔCT method was applied to the data analysis. Relative expression was analyzed using the comparative Ct method.

**Table 3:** Primers used in qRT-PCR

Gene name	Sequences
mMito	Forward: 5’-CTAGAAACCCCGAAACCAAA-3’
	Reverse: 5’-CCAGCTATCACCAAGCTCGT-3’
mDloop	Forward: 5’-AATCTACCATCCTCCGTGAAACC-3’
	Reverse: 5’-TCAGTTTAGCTACCCCCAAGTTTAA-3’
mCol	Forward: 5’-GCCCCAGATATAGCATTCCC-3’
	Reverse: 5’-GTTCATCCTGTTCCTGCTCC-3’
m18S rDNA	Forward: 5’-TAGAGGGACAAGTGGCGTTC-3’
	Reverse: 5’-CGCTGAGCCAGTCAGTGT-3’

### MtDNA extraction and injection

Mitochondrial DNA was extracted from C57BL/6 mouse livers using the mtDNA Isolation Kit (Abcam, #ab65321) following the manufacturer’s instructions. The concentration and purity of isolated mtDNA were confirmed using a NanoDrop 2000. For in vivo transfection, mtDNA was mixed with the transfection reagent Entranster™-in vivo (Engreen Biosystem, #18668-11-2). The control group received Entranster™-in vivo without mtDNA.

### Immunohistochemistry (IHC) analysis

Paraffin-embedded lung sections were deparaffinized, rehydrated, and subjected to antigen retrieval using 10 mM citrate buffer (pH 6.0). Permeabilization was performed using 0.3% Triton X-100. Sections were blocked with 5% goat serum and incubated overnight at 4℃ with anti-P-MLKL antibodies. After PBS washes, a biotinylated secondary antibody was applied and incubated at room temperature for 1 h. Color development was achieved using a DAB substrate kit, followed by PBS washing, dehydration, and sealing. Stained sections were analyzed under a light microscope.

### Immunofluorescence analysis

Paraffin-embedded lung tissue sections were deparaffinized, rehydrated, and subjected to antigen retrieval by boiling in citrate buffer for 10 min. Sections were then permeabilized with 0.3% Triton X-100 and blocked with 5% goat serum. Primary anti-P-TBK1 antibodies were applied and incubated overnight at 4℃. After PBS washing, sections were incubated with a fluorescence-conjugated secondary antibody for 1 h at room temperature. Nuclei were counterstained with 4’,6-diamidino-2-phenylindole (DAPI), and fluorescence images were captured using a fluorescence microscope.

### TUNEL staining

Lung tissue apoptosis was assessed using a fluorescent TUNEL assay kit according to the manufacturer’s instructions. Briefly, paraffin sections were deparaffinized, rehydrated, and washed twice with PBS. TUNEL reaction mixture (50 µL) was applied, and sections were incubated at 37℃ for 60 min in the dark. Nuclei were counterstained with DAPI, and fluorescence signals were observed under a fluorescence microscope.

### Western blotting

Lung tissue samples were flash-frozen in liquid nitrogen and lysed in RIPA buffer containing protease and phosphatase inhibitors. The lysates were centrifuged at 13,000 × g for 15 min, and the supernatants were collected. Protein concentration was determined using a BCA protein assay kit. Equal amounts of protein were separated on 8–12% SDS-PAGE gels and transferred onto PVDF membranes. Membranes were blocked with 5% non-fat milk in TBS containing 0.1% Tween-20 for 1 h at room temperature, then incubated with primary antibodies overnight at 4℃. After washing, membranes were incubated with HRP-conjugated secondary antibodies for 1 h at room temperature. Protein bands were visualized using an enhanced chemiluminescence (ECL) detection system, and densitometric analysis was performed using Quantity One software (Bio-Rad).

### Statistical analysis

Quantitative data are presented as mean ± standard deviation (SD) unless otherwise specified. Data normality was assessed using the Shapiro-Wilk test. Statistical comparisons between two groups were performed using Student’s t-test, while comparisons among multiple groups were analyzed using one-way analysis of variance (ANOVA). A p-value < 0.05 was considered statistically significant. All statistical analyses were conducted using GraphPad Prism 9.0.

## Results

### An increase in circulating MtDNA is associated with inflammation and necroptosis in septic ALI

**Fig. 1:**
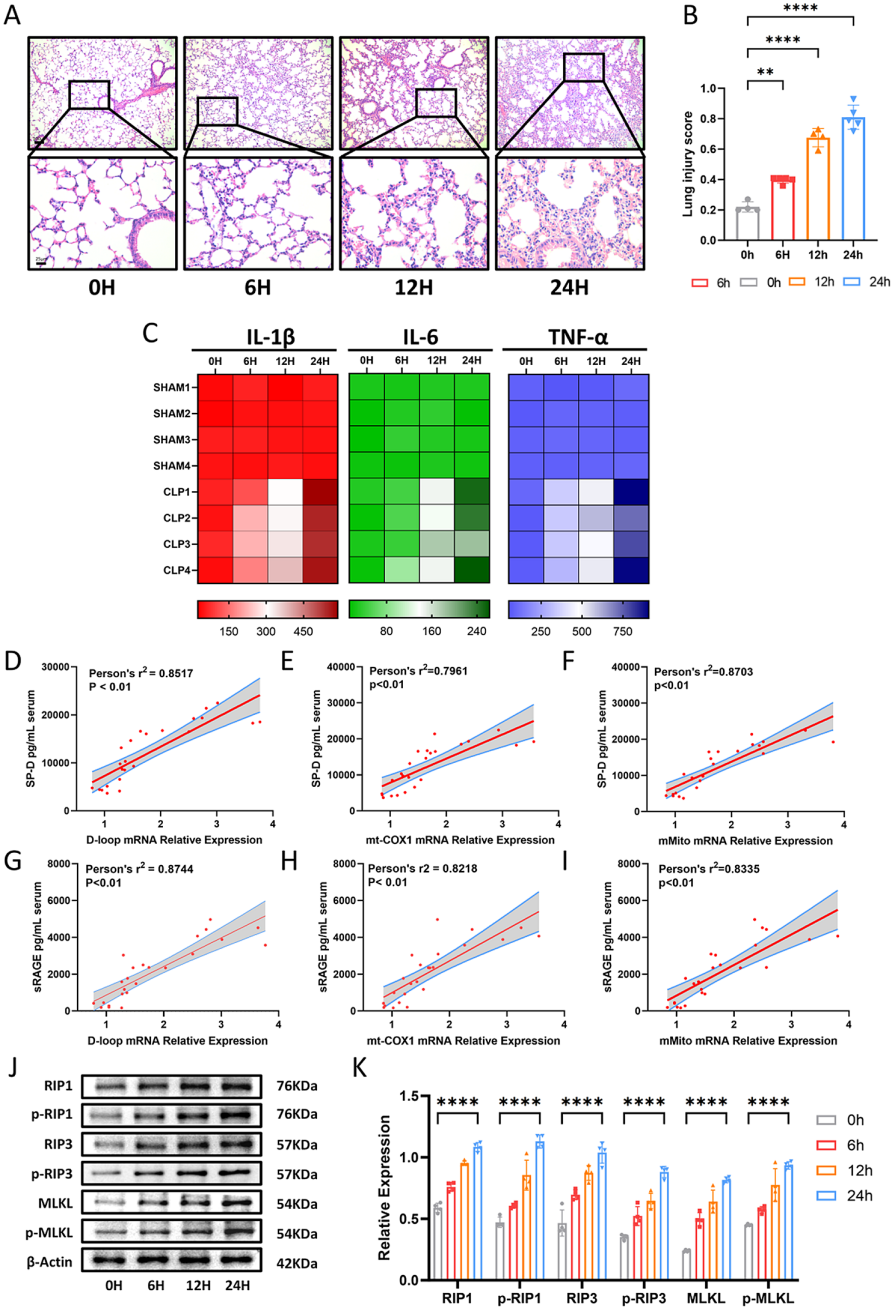
An Increase in Circulating mtDNA Is Associated with Inflammation and Necroptosis in Septic ALI. (**A**) Representative H&E-stained photomicrographs of murine lung tissues from the control (0 h), 6 h, 12 h, and 24 h groups. Bar = 25 μm (original magnification ×100, ×400). (**B**) Lung injury scores for the control (0 h), 6 h, 12 h, and 24 h groups. (**C**) Plasma levels of inflammatory cytokines (IL-1β, IL-6, and TNF-α) measured by ELISA in mice after CLP at 0, 6, 12, and 24 h. (**D-I**) Correlations between serum mtDNA levels and lung injury biomarkers (SP-D and sRAGE) assessed using the Spearman correlation test. mtDNA levels were analyzed via quantitative real-time PCR using three mtDNA primers (D-loop, mt-COX1, mMito). (**J**) Western blot analysis of necroptosis signaling in murine lung tissues at 0, 6, 12, and 24 h after CLP. (**K**) Quantification of Western blot results. Data are presented as mean ± SD from at least four independent experiments. *p < 0.05; **p < 0.01; ***p < 0.001; ****p < 0.0001; ns, not significant. One-way ANOVA followed by Tukey’s multiple comparisons test

To assess the severity of lung injury over time following CLP, HE staining was performed (Fig. 1A, B). The results showed a progressive increase in lung injury scores, characterized by interstitial thickening, immune cell infiltration, and alveolar fluid accumulation. The observed increase in immune cell infiltration suggested an intensifying inflammatory response. ELISA analysis confirmed this by demonstrating a time-dependent rise in serum inflammatory cytokines, including IL-1β, IL-6, and TNF-α, after CLP (Fig. 1C). Given that circulating mtDNA has been identified as a potential predictor of lung disease and sepsis prognosis, we investigated its relationship with inflammatory markers of lung injury. As expected, mtDNA levels gradually increased following CLP and exhibited a positive correlation with lung injury markers (Fig. 1D-I). Additionally, protein expression analysis of necroptosis-related pathways in septic lung tissues (Fig. 1J, K) revealed a progressive elevation in necroptosis-associated proteins over time. These findings indicate that CLP-induced sepsis leads to the release of circulating mtDNA, accompanied by necroptosis activation and an upregulation of inflammatory markers in lung tissues.

### Melatonin mitigates septic ALI by reducing Circulating MtDNA release and inflammatory injury

**Fig. 2:**
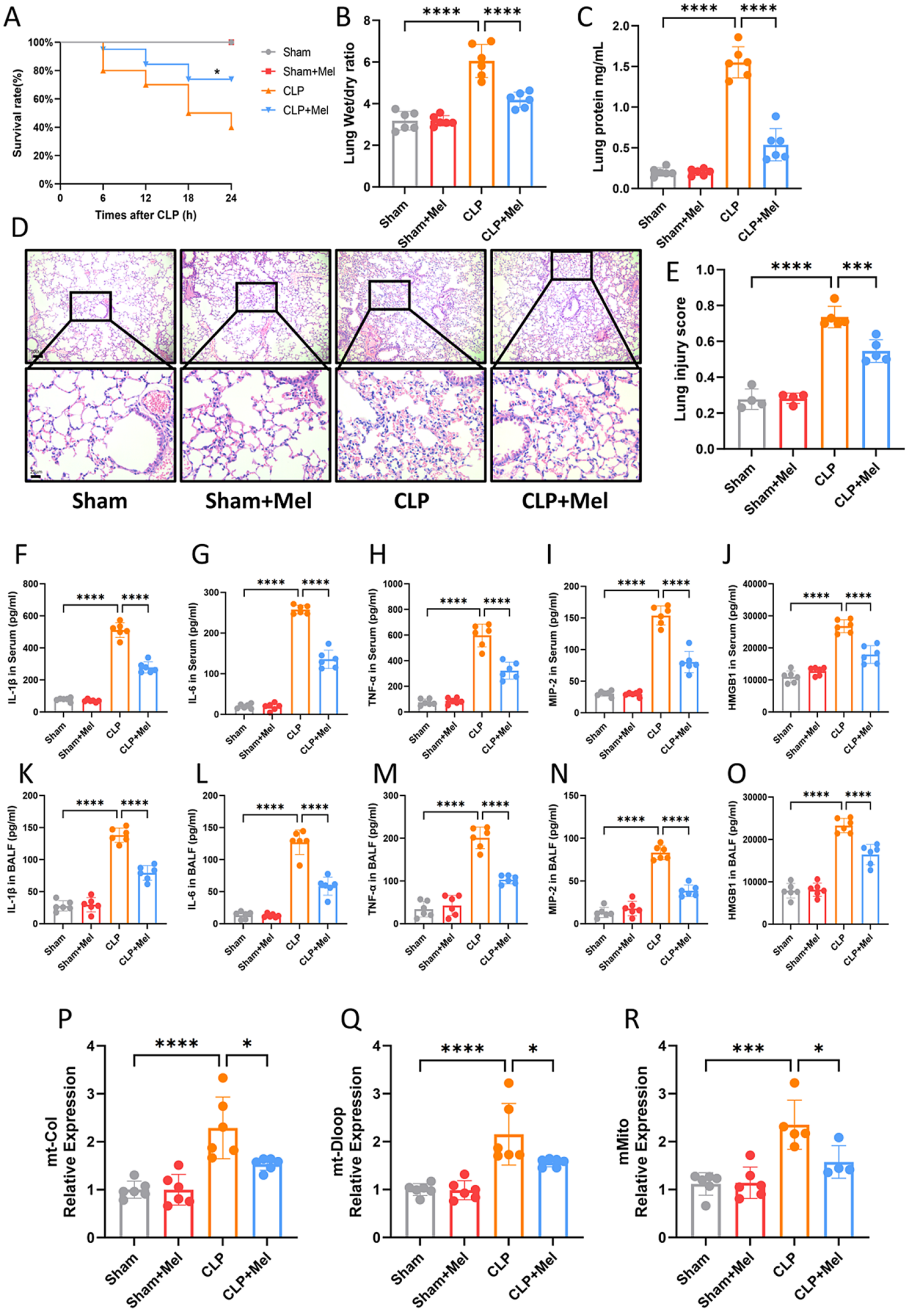
Melatonin Mitigates Septic ALI by Reducing Circulating mtDNA Release and Inflammatory Injury. (**A**) Survival rates of mice with or without melatonin therapy 24 h after CLP (log-rank [Mantel-Cox] test, *n* = 20). (**B**) Lung wet/dry ratio in mice with or without melatonin therapy at 24 h after CLP. (**C**) BALF protein levels in mice with or without melatonin therapy at 24 h after CLP. (**D**) Representative H&E-stained photomicrographs of lung tissues from Sham, Sham + Mel, CLP, and CLP + Mel groups. Bar = 25 μm (original magnification ×100, ×400). (**E**) Lung injury scores from the Sham, Sham + Mel, CLP, and CLP + Mel groups. (**F-J**) Plasma levels of inflammatory cytokines (IL-1β, IL-6, TNF-α, MIP-2, and HMGB1) measured by ELISA in Sham, Sham + Mel, CLP, and CLP + Mel groups. (**K-O**) BALF levels of inflammatory cytokines (IL-1β, IL-6, TNF-α, MIP-2, and HMGB1) measured by ELISA in the same groups. (**P-R**) qPCR analysis of mt-Col, mt-Dloop, and mMito mRNA levels in indicated tissues. Data are presented as mean ± SD. *p < 0.05; **p < 0.01; ***p < 0.001; ****p < 0.0001; ns, not significant. One-way ANOVA followed by Tukey’s multiple comparisons test

To explore the protective effects of melatonin, CLP mice were treated with melatonin, which resulted in a significant reduction in mortality and lung injury scores. This was evidenced by a decrease in pulmonary edema and reduced protein infiltration in the pulmonary interstitium (Fig. 2A, B, C). HE staining confirmed that melatonin treatment alleviated CLP-induced lung injury (Fig. 2D, E). Further analysis of inflammatory cytokines in serum and BALF showed that CLP led to increased levels of inflammatory mediators, which were significantly reduced following melatonin treatment (Fig. 2F-O). These findings suggest that melatonin exerts a protective effect by suppressing inflammatory responses in both the circulatory system and lung tissues. Additionally, serum mtDNA levels were assessed, revealing that melatonin markedly reduced mtDNA release and inflammatory cytokine production triggered by CLP (Fig. 2P, Q, R). These results suggest that the protective effects of melatonin may be attributed to its ability to mitigate inflammation and suppress mtDNA release.

### Melatonin alleviates Sepsis-Induced ALI by inhibiting RIP1/RIP3/MLKL-Mediated necroptosis

**Fig. 3:**
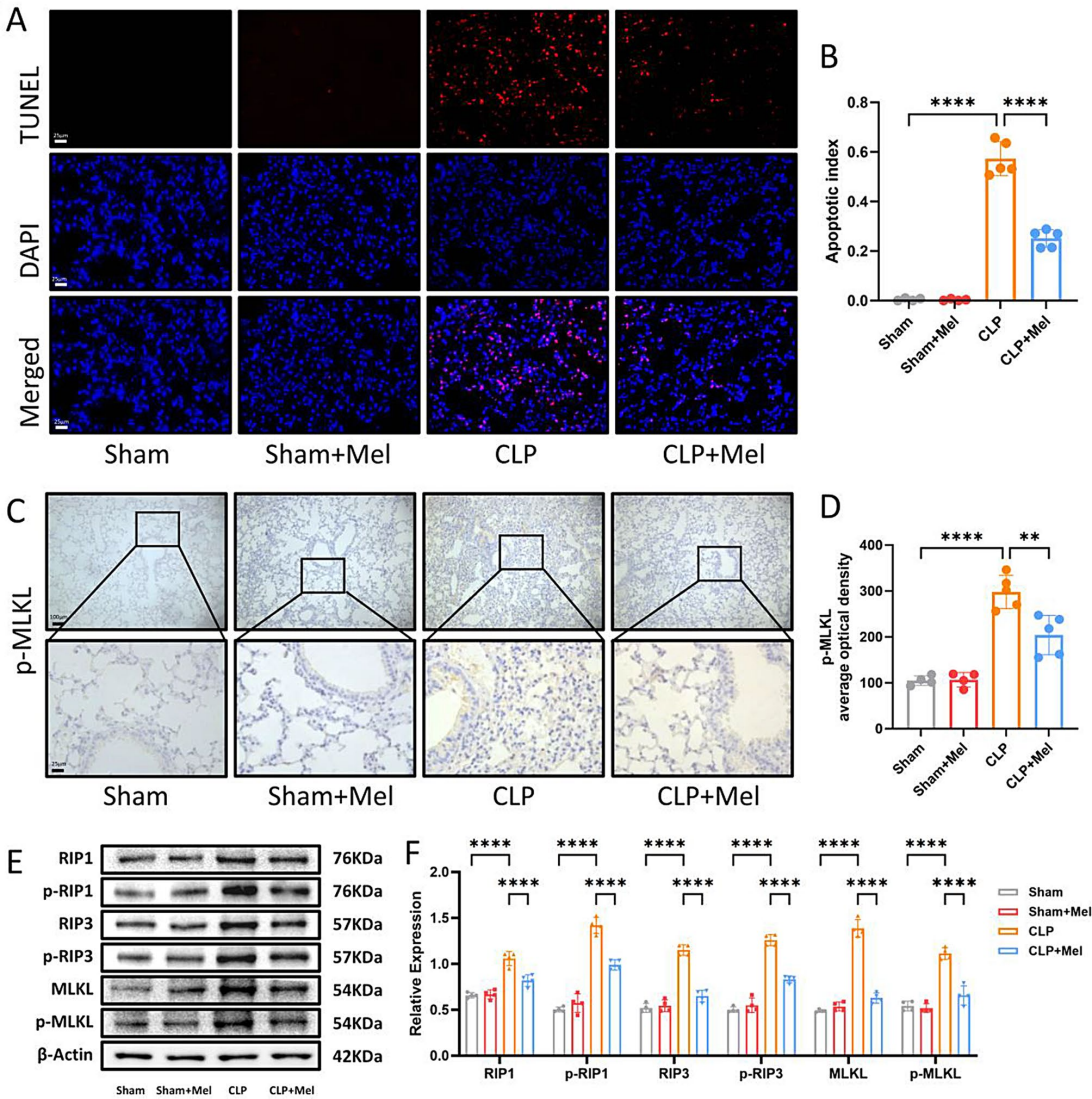
Melatonin Alleviates Sepsis-Induced ALI by Inhibiting RIP1/RIP3/MLKL-Mediated Necroptosis. (**A**) TUNEL staining analysis of cell death in Sham, Sham + Mel, CLP, and CLP + Mel groups. Bar = 25 μm (original magnification ×400). (**B**) Apoptotic index represented by the ratio of positive cells to DAPI-stained cells. (**C**) Representative images of IHC analysis for p-MLKL expression in lung tissues. (**D**) Quantification of p-MLKL optical density in immunohistochemistry using ImageJ. Bar = 25 μm (original magnification ×100, ×400). (**E**) Western blot analysis of necroptosis signaling in the lungs of Sham, Sham + Mel, CLP, and CLP + Mel groups. (**F**) Quantification of Western blot results. Data are presented as mean ± SD from at least four independent experiments. *p < 0.05; **p < 0.01; ***p < 0.001; ****p < 0.0001; ns, not significant. One-way ANOVA followed by Tukey’s multiple comparisons test

To investigate the potential mechanisms underlying melatonin’s protective effects in ALI, TUNEL staining was used to assess the level of cell death in lung tissue. Immunofluorescence analysis showed that CLP induced significant cell death in lung tissues, which was significantly reduced by melatonin treatment (Fig. 3A, B). To determine the specific mode of death, IHC was employed to access p-MLKL expression. IHC staining further demonstrated that melatonin administration decreased p-MLKL expression in lung tissues, indicating that melatonin attenuated the occurrence of necroptosis (Fig. 3C, D). Western blot analysis revealed that CLP induced an upregulation of necroptosis-related proteins, including P-RIP1, RIP1, P-RIP3, RIP3, P-MLKL, and MLKL. However, these increases were significantly attenuated following melatonin treatment (Fig. 3E, F). These findings suggest that melatonin protects against CLP-induced ALI by inhibiting RIP1/RIP3/MLKL-mediated necroptosis.

### Circulating MtDNA induces ALI in CLP mice by activating RIP1/RIP3/MLKL-Mediated necroptosis

**Fig. 4:**
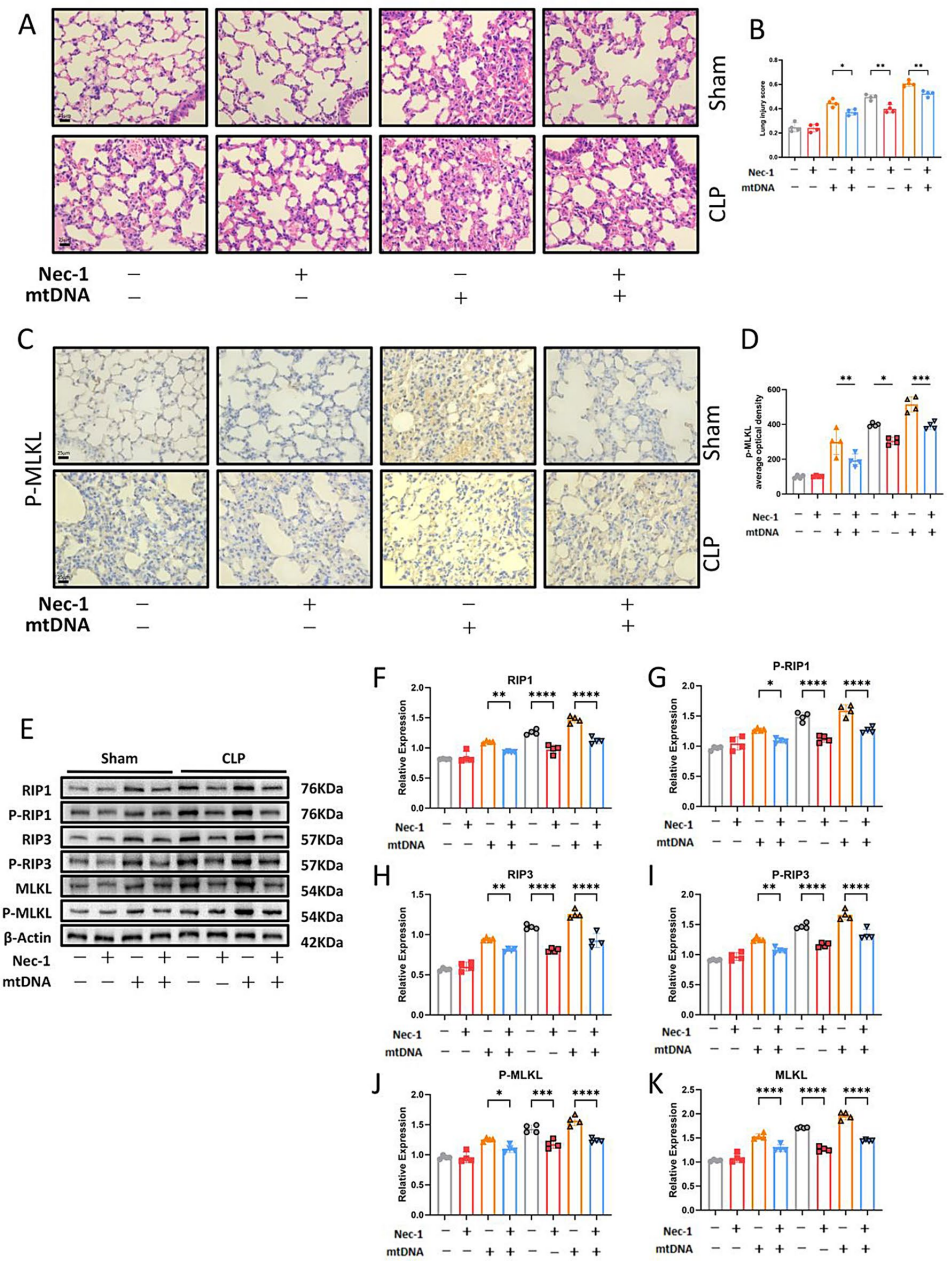
Circulating mtDNA Induces ALI in CLP Mice by Activating RIP1/RIP3/MLKL-Mediated Necroptosis. (**A**) Representative H&E-stained photomicrographs of lung tissues from Sham and CLP groups, with or without Nec-1 and mtDNA injection. Bar = 25 μm (original magnification ×400). (**B**) Lung injury scores in Sham and CLP groups, with or without Nec-1 and mtDNA injection. (**C**) Representative IHC images for p-MLKL expression in lung tissues. (**D**) Quantification of p-MLKL optical density in immunohistochemistry using ImageJ. Bar = 25 μm (original magnification ×400). (**E**) Western blot analysis of necroptosis signaling in the lungs of Sham and CLP groups, with or without Nec-1 and mtDNA injection. (**F-G**) Quantification of Western blot results. Data are presented as mean ± SD from at least four independent experiments. *p < 0.05; **p < 0.01; ***p < 0.001; ****p < 0.0001; ns, not significant. One-way ANOVA followed by Tukey’s multiple comparisons test

Since mtDNA levels were positively correlated with inflammatory responses and necroptosis in lung tissues, we hypothesized that mtDNA release might contribute to ALI by triggering necroptosis in CLP mice. To further explore this mechanism, mice were administered isolated mtDNA along with Nec-1, a specific RIP inhibitor, via intraperitoneal injection. HE staining revealed that mtDNA administration exacerbated lung injury, while Nec-1 treatment significantly reversed these effects (Fig. 4A, B). IHC staining showed that mtDNA administration led to increased p-MLKL expression, which was similarly mitigated by Nec-1 treatment (Fig. 4C, D). Western blot analysis further confirmed that mtDNA injection significantly upregulated necroptosis-related proteins, and these effects were reversed by Nec-1 administration (Fig. 4E-K). These results indicate that circulating mtDNA induces ALI in CLP mice by activating RIP1/RIP3/MLKL-mediated necroptosis.

### Melatonin alleviates STING pathway activation after Sepsis

**Fig. 5:**
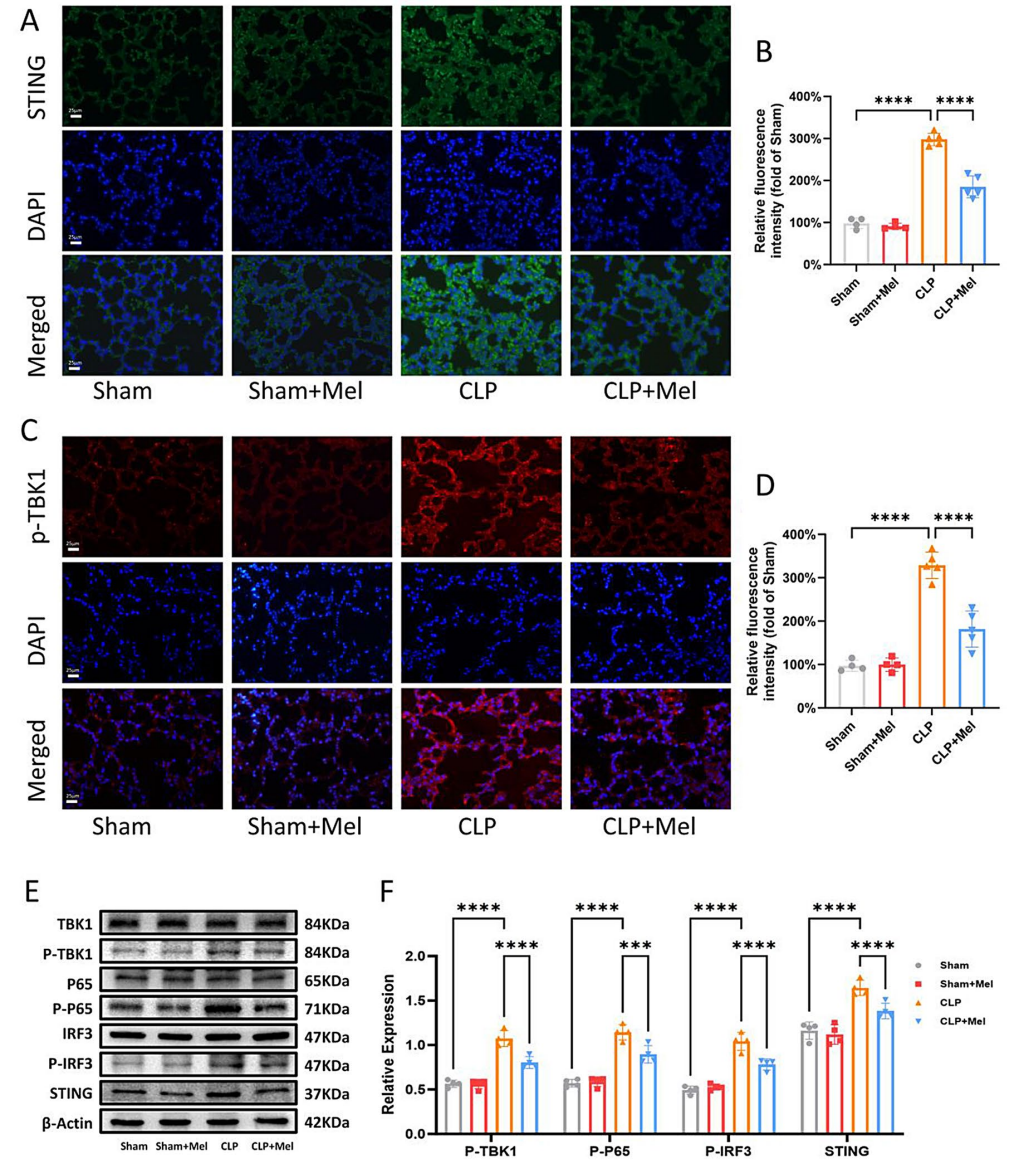
Melatonin Alleviates STING Pathway Activation After Sepsis. (**A**) Immunofluorescence staining of STING in lung tissues of mice. Green, STING immunostaining; blue, DAPI-stained DNA. Scale bar = 25 μm (original magnification ×400). (**B**) Quantification of relative fluorescence intensity for STING. (**C**) Immunofluorescence staining of p-TBK1 in lung tissues of mice. Red, p-TBK1 immunostaining; blue, DAPI-stained DNA. Scale bar = 25 μm (original magnification ×400). (**D**) Quantification of relative fluorescence intensity for p-TBK1. (**E**) Western blot analysis of STING signaling in the lungs of Sham, Sham + Mel, CLP, and CLP + Mel groups. (**F**) Quantification of Western blot results. Data are presented as mean ± SD from at least four independent experiments. *p < 0.05; **p < 0.01; ***p < 0.001; ****p < 0.0001; ns, not significant. One-way ANOVA followed by Tukey’s multiple comparisons test

In this study, we demonstrated that melatonin treatment significantly alleviates septic ALI by reducing mtDNA release and necroptosis. Since released mtDNA can activate the STING signaling pathway and enhance the expression of inflammatory factors, we hypothesized that melatonin reduces circulating mtDNA levels, thereby inhibiting STING pathway activation in lung tissues and alleviating ALI. To test this hypothesis, we examined the expression of STING-related proteins. Immunofluorescence analysis revealed that CLP increased the expression of STING protein and its downstream effector, phosphorylated TBK1 (P-TBK1), both of which were significantly attenuated by melatonin treatment (Fig. 5A-D). Western blot analysis further confirmed that melatonin reduced the activation of the STING pathway, as evidenced by decreased expression levels of STING, phosphorylated IRF3 (p-IRF3), phosphorylated TBK1 (p-TBK1), and phosphorylated p65 (p-p65). These findings suggest that melatonin inhibits STING pathway activation, thereby exerting an anti-inflammatory effect.

### The mtDNA-STING signaling pathway promotes ALI development by activating necroptosis

As previously demonstrated, melatonin reduces necroptosis and inhibits STING activation induced by circulating mtDNA release. To further investigate the relationship between STING signaling and necroptosis, we administered septic mice with either a STING agonist or inhibitor in addition to melatonin treatment and analyzed changes in necroptosis-related markers.

**Fig. 6:**
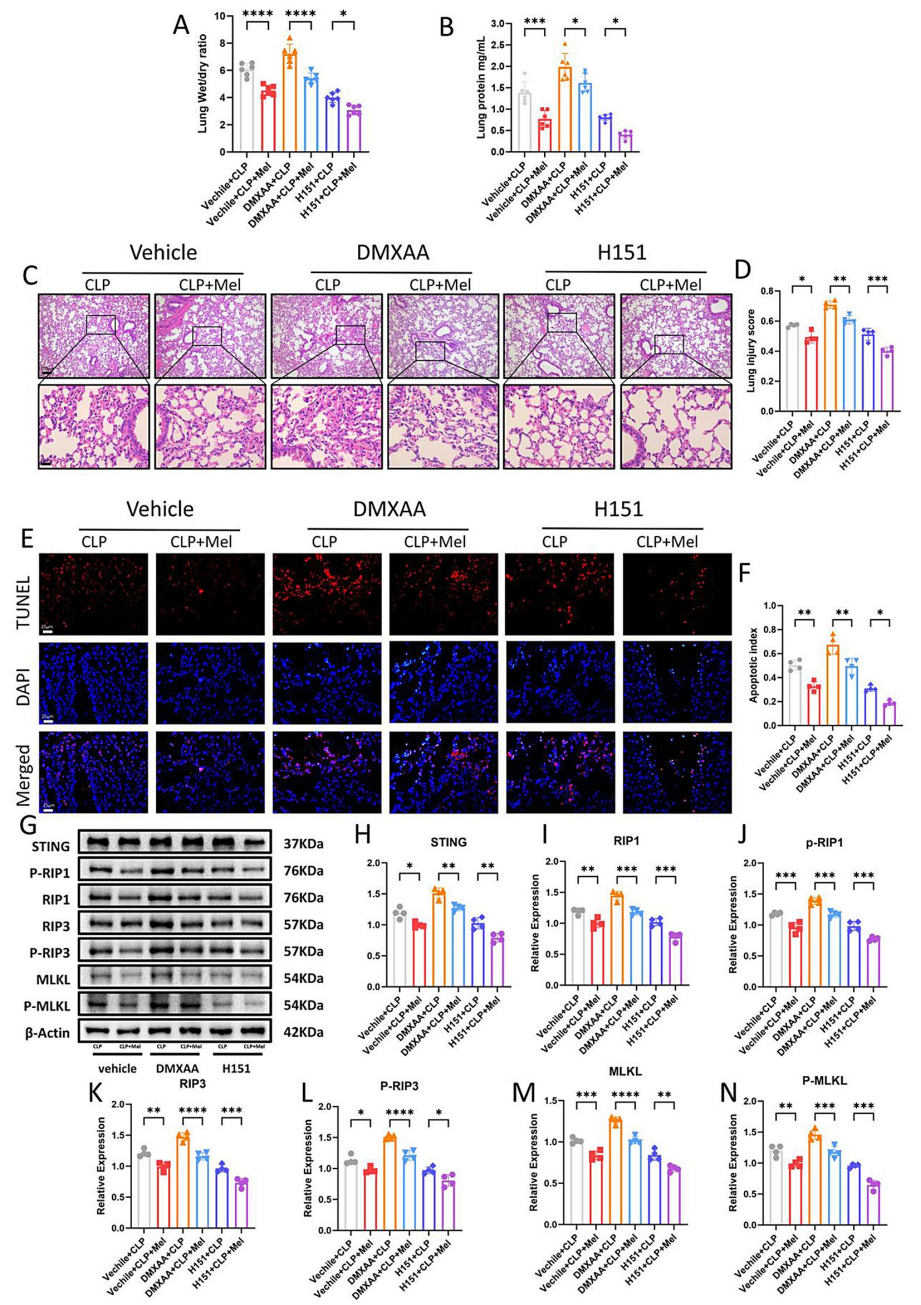
The mtDNA-STING Signaling Pathway Promotes ALI Development by Activating Necroptosis. (**A**) Lung wet/dry ratio in mice with or without melatonin therapy at 24 h after CLP. (**B**) BALF protein levels in mice with or without melatonin therapy at 24 h after CLP. (**C**) Representative H&E-stained photomicrographs of lung tissues from Vehicle, DMXAA, and H151 groups, with or without melatonin therapy. Bar = 25 μm (original magnification ×100, ×400). (**D**) Lung injury scores in the Vehicle, DMXAA, and H151 groups, with or without melatonin therapy. (**E**) TUNEL staining analysis of cell death in Vehicle, DMXAA, and H151 groups, with or without melatonin therapy. Bar = 25 μm (original magnification ×400). (**F**) Apoptotic index represented by the ratio of positive cells to DAPI-stained cells. (**G**) Western blot analysis of STING signaling in lung tissues of Vehicle, DMXAA, and H151 groups, with or without melatonin therapy. (**H-N**) Quantification of Western blot results. Data are presented as mean ± SD from at least four independent experiments. *p < 0.05; **p < 0.01; ***p < 0.001; ****p < 0.0001; ns, not significant. One-way ANOVA followed by Tukey’s multiple comparisons test

Following administration of the STING agonist DMXAA, we observed exacerbated pulmonary pathological injury, as indicated by worsening pulmonary edema and increased pulmonary interstitial protein infiltration (Fig. 6A, B). HE staining and lung injury scores indicated that melatonin and H151 treatment attenuated lung injury (Fig. 6C, D). Subsequently, we used TUNEL staining to determine the degree of lung cell death. Our results expressed that melatonin reversed lung cell death induced by DMXAA, suggesting that melatonin may act by inhibiting STING activation (Fig. 6E, F). Then, we detected changes in STING proteins and necroptosis related pathway proteins. Western blot analysis revealed that STING pathway activation and necroptosis-related protein expression were significantly upregulated following DMXAA stimulation. However, these increases were substantially attenuated by either H151 or melatonin treatment (Fig. 6G-N). These findings indicate that melatonin mitigates necroptosis by inhibiting mtDNA release and, consequently, suppressing STING pathway activation, thereby preventing the progression of ALI.

## Discussion

**Fig. 7:**
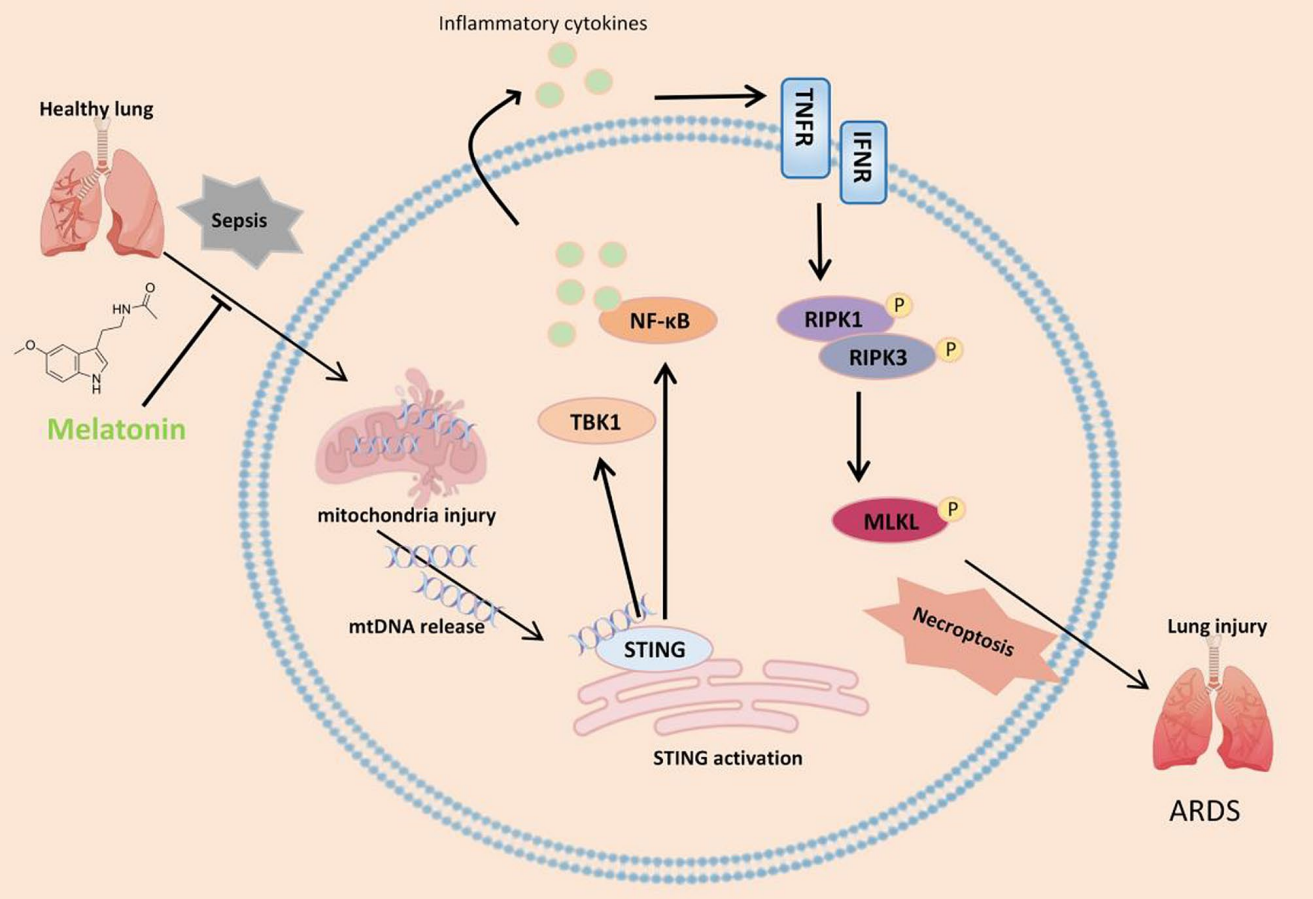
Schematic illustration of this study. Melatonin reduces mtDNA leakage, thereby inhibiting STING activation, suppressing necroptosis, and ultimately mitigating lung injury and improving lung function

Sepsis is a leading cause of ARDS, and melatonin has been shown to alleviate ARDS-related injury by reducing inflammatory activation (Kang et al. 2022). However, the mechanisms underlying sepsis-induced necroptosis in ARDS and the regulatory role of melatonin in lung cell necroptosis and inflammation remain largely unclear. In this study, we elucidated the mechanism of lung injury in sepsis-induced ARDS and identified melatonin as a potent protective agent that prevents ARDS by inhibiting necroptosis. Our findings demonstrate that STING signaling is activated in the lungs of septic mice due to mtDNA release, which subsequently triggers RIPK1/RIPK3/MLKL-mediated necroptosis, initiating a cascade of inflammatory responses. Melatonin effectively reduces mtDNA leakage, thereby inhibiting STING activation, suppressing necroptosis, and ultimately mitigating lung injury and improving lung function. These findings provide novel molecular insights into potential therapeutic targets for sepsis-induced ARDS (Fig. 7).

Despite advancements in modern medicine, effective treatment for sepsis remains limited, and its morbidity and mortality rates remain high (Poll et al. 2017). The lungs are particularly susceptible to sepsis-induced injury(Martin et al. 2003). The excessive immune response triggered by sepsis leads to pulmonary capillary endothelial and alveolar epithelial edema and exudation, resulting in the breakdown of the alveolar-capillary barrier. This disruption reduces effective ventilation, ultimately impairing lung function and posing a severe threat to patient survival (Park et al. 2019; Huo et al. 2023). During ARDS progression, cellular stress and immune-mediated damage release DAMPs, leading to mitochondrial dysfunction and subsequent macrophage activation, which promotes the release of inflammatory cytokines and chemokines (Bos and Ware 2022; Gorman et al. 2022). Our study confirmed that inflammatory cytokines, including IL-1β, IL-6, and TNF-α, are positively correlated with lung injury severity, and lung injury markers such as SP-D and sRAGE are also elevated during ALI progression. These findings highlight the pivotal role of inflammation in ALI and underscore the importance of identifying inflammation-induced mechanisms to develop effective treatments.

Necroptosis, a regulated form of cell death, is primarily mediated by RIPK1, RIPK3, and MLKL, leading to cell swelling, organelle dysfunction, and plasma membrane rupture (Ju et al. 2022; Weinlich et al. 2017; Kolbrink et al. 2023). Previous studies have identified RIPK3-mediated necroptosis as a key mechanism in acute lung injury (Yang et al. 2022). Additionally, RIPK3 and MLKL gene expression in circulating leukocytes has been positively correlated with sepsis-related mortality, further emphasizing the role of necroptosis in sepsis-induced lung injury (Liu et al. 2021a). Our findings demonstrate that as sepsis progresses, serum inflammatory markers and necroptosis-related protein expression increase, suggesting that necroptosis is closely associated with inflammation and contributes to ALI progression.

Melatonin has gained attention in recent years due to its diverse physiological functions, including regulation of inflammatory factors, oxidative stress reduction, and free radical scavenging (Sun et al. 2015; Sagrillo-Fagundes et al. 2018; Zhang et al. 2022). Numerous studies have reported that melatonin exerts anti-inflammatory effects (Wang et al. 2023; Hardeland 2018). Our study confirmed that melatonin reduces serum inflammatory cytokine levels in septic mice, which is consistent with previous studies (Yu et al. 2020). Previous research on ARDS has primarily focused on melatonin’s protective effects in reducing oxidative stress and inflammasome activation (Kang et al. 2022; Zheng et al. 2023; Zhang et al. 2016). Recent metabolomic and transcriptomic studies have suggested that melatonin may play a protective role in COPD by inhibiting necroptosis (Mao et al. 2021). However, the role of melatonin in mitigating necroptosis in ARDS and its precise mechanism remain unclear. Our study revealed that melatonin reduces necroptosis marker expression in lung tissues, further supporting its protective role in sepsis-induced lung injury.

Mitochondria are central to cellular energy metabolism and play a critical role in maintaining cellular stability and function (Nunnari and Suomalainen 2012). Inflammatory cytokines, chemokines, and DAMPs generated during inflammation compromise mitochondrial membrane integrity, leading to mitochondrial damage, dysfunction, and subsequent release of mitochondrial DNA (mtDNA) into the cytoplasm (Marchi et al. 2023; Zhang et al. 2010; Todkar et al. 2021; Tumburu et al. 2021; Vringer and Tait 2023). Increasing evidence suggests that mtDNA release is associated with necroptosis induction (Liu et al., 2021b; Zhu et al. 2021; Chen et al. 2018). Studies have shown that cytoplasmic mtDNA activates signaling pathways, such as the calpain/CDK5/Drp1 axis, to promote MLKL-mediated necroptosis in neurons (Qiang et al. 2023). Additionally, mtDNA-STING interaction has been implicated in intestinal ischemia-reperfusion injury by coordinating IFN and TNF-α signaling (Zhang et al. 2020). Given the strong association between mtDNA and necroptosis, we further investigated this relationship by administering intraperitoneal mtDNA injections in mice while simultaneously using necroptosis inhibitors. Our results confirm that mtDNA directly induces lung necroptosis.

The cyclic GMP-AMP synthase (cGAS)-STING pathway is a crucial intracellular immune signaling pathway involved in cytoplasmic DNA-mediated immune responses(Hopfner and Hornung 2020; Decout et al. 2021; Gulen et al. 2023). It plays a key role in various inflammatory diseases by inducing proinflammatory cytokines. The cGAS-STING pathway has emerged as a central mediator of inflammation in infection, cellular stress, and tissue injury (Wang et al., 2020b). Upon sensing endogenous DNA, such as mtDNA, cGAS binds to dsDNA, activates STING, and triggers an inflammatory cascade (Samson and Ablasser 2022). To further investigate whether melatonin mitigates inflammation by suppressing the STING pathway, we examined changes in STING signaling. Our results demonstrate that melatonin significantly inhibits STING activation, suggesting that its anti-inflammatory effects are mediated through STING suppression.

Rojas-Rivera et al. reported that inhibitors of protein kinase R-like ER kinase (PERK), a key catalyst of the STING pathway, were effective in inhibiting RIPK1, suggesting a potential link between STING signaling and necroptosis (Rojas-Rivera et al. 2017). Additionally, Brault et al. demonstrated that the cGAS-STING pathway triggers necroptosis in primary macrophages when caspase activity is suppressed, further elucidating the relationship between STING and necroptosis (Brault et al. 2018), In our study, we observed that the release of mtDNA leads to STING activation and necroptosis in septic ARDS. Our results indicate that mtDNA induces the activation of the STING pathway, which subsequently triggers lung necroptosis. Melatonin was able to inhibit the release of mtDNA and reduce STING pathway activation, ultimately mitigating necrotic injury in sepsis-induced ALI.

There are some limitations in our study that should be acknowledged. While we found that melatonin alleviates necrosis by reducing the mtDNA-induced inflammatory response, the exact relationship between this mechanism and melatonin’s anti-inflammatory and antioxidant effects remains unclear. Although we have explored the role of sepsis-induced lung cell necrosis, our understanding of the interactions between various modes of cell death is still limited. Future studies will aim to further investigate the mechanisms underlying sepsis-mediated injury. Additionally, although our findings suggest that STING activation induces necroptosis, some studies have indicated that the RIPK3-MLKL necroptosis pathway can amplify STING signaling, aggravating sepsis progression (Zhang et al. 2023). This suggests that the relationship between STING signaling and necroptosis may not be purely upstream-downstream, and further research is required to clarify the specific mechanisms involved.

## Conclusion

Our study reveals that targeting mtDNA-mediated necroptosis may serve as a novel therapeutic strategy to reduce sepsis-associated lung injury. Furthermore, our findings provide mechanistic support for the clinical use of melatonin in sepsis treatment, highlighting its potential as a promising therapeutic agent.

## Data Availability

No datasets were generated or analysed during the current study.
